# *Toxoplasma gondii* Mechanisms of Entry Into Host Cells

**DOI:** 10.3389/fcimb.2020.00294

**Published:** 2020-06-30

**Authors:** Juliana Portes, Emile Barrias, Renata Travassos, Márcia Attias, Wanderley de Souza

**Affiliations:** ^1^Laboratório de Ultraestrutura Celular Hertha Meyer, Instituto de Biofísica Carlos Chagas Filho, Universidade Federal Do Rio de Janeiro, Rio de Janeiro, Brazil; ^2^Centro Nacional de Biologia Estrutural e Bioimagem, Instituto Nacional de Ciência e Tecnologia em Biologia Estrutural e Bioimagem, Rio de Janeiro, Brazil; ^3^Laboratório de Metrologia Aplicada à Ciências da Vida, Instituto Nacional de Metrologia, Qualidade e Tecnologia- Inmetro, Rio de Janeiro, Brazil

**Keywords:** endocytosis, clathrin, macropinocytosis, *Toxoplasma gondii*, host cell

## Abstract

*Toxoplasma gondii*, the causative agent of toxoplasmosis, is an obligate intracellular protozoan parasite. *Toxoplasma* can invade and multiply inside any nucleated cell of a wide range of homeothermic hosts. The canonical process of internalization involves several steps: an initial recognition of the host cell surface and a sequential secretion of proteins from micronemes followed by rhoptries that assemble a macromolecular complex constituting a specialized and transient moving junction. The parasite is then internalized via an endocytic process with the establishment of a parasitophorous vacuole (PV), that does not fuse with lysosomes, where the parasites survive and multiply. This process of host cell invasion is usually referred to active penetration. Using different cell types and inhibitors of distinct endocytic pathways, we show that treatment of host cells with compounds that interfere with clathrin-mediated endocytosis (hypertonic sucrose medium, chlorpromazine hydrochloride, and pitstop 2 inhibited the internalization of tachyzoites). In addition, treatments that interfere with macropinocytosis, such as incubation with amiloride or IPA-3, increased parasite attachment to the host cell surface but significantly blocked parasite internalization. Immunofluorescence microscopy showed that markers of macropinocytosis, such as the Rab5 effector rabankyrin 5 and Pak1, are associated with parasite-containing cytoplasmic vacuoles. These results indicate that entrance of *T. gondii* into mammalian cells can take place both by the well-characterized interaction of parasite and host cell endocytic machinery and other processes, such as the clathrin-mediated endocytosis, and macropinocytosis.

## Introduction

Intracellular parasitism is a strategy adopted by several eukaryotic microorganisms that developed the ability to penetrate and divide inside cells of vertebrate hosts. Some have a narrow choice of specific cell types in which they can penetrate, as is the case for *Plasmodium* and *Leishmania*, that infect reticulocytes-erythrocytes and macrophages, respectively (Podinovskaia and Descoteaux, 2015; Kanjee et al., 2018). Other parasites, such as *Trypanosoma cruzi* and *Toxoplasma gondii*, can invade almost all cell types (Zhu et al., 2019; Breyner et al., 2020). It is common sense that invasion of host is advantageous in at least two important aspects: isolation from host immune response and because it allows the access to nutrients from the host cell. Studies carried out with several intracellular microorganisms show that all are internalized through an endocytic process that results in a membrane-bound vacuole inside the host cell, the parasitophorous vacuole (PV). Some organisms survive and multiply within this vacuole, as occurs with *T. gondii*, while others disrupt the parasitophorous vacuole membrane and then have direct contact with the host cytoplasm (Walker et al., 2014; Guérin et al., 2017). Microorganisms may take advantage of several endocytic processes already described, including phagocytosis, macropinocytosis and clathrin-mediated endocytosis (reviewed in Mayor and Pagano, 2007; Mayor et al., 2014) to enter host cells. Previous studies shown that *T. gondii* has an active participation in the penetration into the host cell, even interfering with the composition of the PV membrane to prevent its fusion with host cell lysosomes (Morisaki et al., 1995; Coppens et al., 2006; Frénal et al., 2017) Organelles from the apical complex of the parasite, as micronemes and rhoptries, release their contents during the interaction process (Dubremetz et al., 1993; Carruthers and Sibley, 1997; Carruthers et al., 1999).

The early stage of the internalization process is initiated with the interaction between *T. gondii* apical end with the surface of the prospective host cell. Adhesion and recognition of surface molecules between the parasite and the future host cell occurs by low affinity bonds with molecules constitutively exposed in the outer surface of the parasite's membrane, anchored to it by glicosyl-phosphatidyl-inositol (GPI). Surface molecules such as SAGs (surface antigens) (reviewed by Carruthers and Boothroyd, 2007) recognize a wide range of receptors in different cells types, such as heparan sulfate, proteoglycans and laminin (Haas and Plow, 1994; Ortega-Barria and Boothroyd, 1999; Carruthers et al., 2000). Proteins specifically secreted by the neck of the rhoptries, RON 2, RON 4 and RON 5 form a complex on the host cell membrane and RON2 has a domain that serves as a receptor for the parasite. The binding of this receptor to AMA1, a protein secreted by micronemes, anchored to the parasite membrane is the basic mechanism by which *T. gondii* recognizes any type of cell (Tonkin et al., 2011). Proteins secreted by micronemes (MICs) that are incorporated to the plasma membrane of the tachyzoite mediate the adhesion between the parasite and the membrane of the host cell. MICs also connects to cytoplasmic domains establishing connections with parasite F-actin that interact with myosin TgMyoA, and the inner membrane complex (IMC) of *T. gondii*. Myosin promotes sliding of actin filaments to the posterior end of the parasite, which displaces the IMC-receptor complex bound to these filaments, resulting in the parasite moving forward toward the endocytic vacuole (reviewed by Daher and Soldati-Favre, 2009). In the early steps of this interaction, signaling culminates in intracellular calcium firing and microneme secretion (reviewed in Blader and Saeij, 2009). Subsequently, rhoptry secretion is induced. Proteins secreted by the parasite, such as AMA1, RON 2, RON 4, RON 5, and RON 8, assemble the moving junction, a dynamic structure, which is crucial for the success of parasite active invasion (Mordue et al., 1999; Sibley, 2011). Some parasites may also be internalized via a typical phagocytic process, especially into macrophages; forming large vacuoles that may fuse with host cell lysosomes with subsequent destruction of the parasite (Walker, 2007; De Souza and De Carvalho, 2013; Lidani et al., 2017).

Endocytosis is described as a set of mechanisms that enable the partial internalization of plasma membrane carrying extracellular fluids, molecules, or particles into the cytoplasm. It has been shown that many pathogens are able to use different endocytic processes to penetrate into host cells (Walker et al., 2014). Recently, endocytic pathways are classified as: classical (clathrin-mediated) endocytosis, caveolin 1-dependent endocytosis (caveolae), CLIC/GEEC-type endocytosis, Arf6-dependent endocytosis, flotillin-dependent endocytosis, phagocytosis, and macropinocytosis. A large diversity of filaments, adapter molecules, and accessory proteins is used for vesicle formation, reflecting the enormous diversity of materials that must be packaged. Some of the known loads utilizing the clathrin-mediated endocytosis pathway are tyrosine kinase receptors, transferrin receptors, LDL receptors, and anthrax toxin (Andersson, 2012). *In vitro* studies suggest that the clathrin structure can accommodate incoming loads with a maximum diameter of 120 nm (Doherty and McMahon, 2009). Clathrin-mediated endocytosis has been documented as a gateway to different viruses, such as influenza virus, Ebola, orthobunyavirus, and hepatitis B, C, and E in different host cells (Blanchard et al., 2006; Cooper and Shaul, 2006; Marsh and Helenius, 2006; Huang et al., 2012). Challenging the dogma that only particles as large as 120 nm can enter clathrin-dependent cells, several studies have shown that bacteria, such as *Listeria monocytogenes, Yersinia pseudotuberculosis, Rickettsieae*, and *Chlamydia trachomatis* and the protozoan *T. cruzi*, may invade cells via this pathway (Barrias et al., 2012; Feng et al., 2018; Latomanski and Newton, 2019). Another strategic pathway of cell entry for parasites is macropinocytosis. Canonically, this pathway consists in the engulfing of significant amounts of extracellular fluid by a large extension of the plasma membrane with fusion of plasma membrane extensions generating vesicles larger than 1 μm (macropinosomes). The formation of macropinosomes is dependent of actin filaments rearrangement (Johannes and Lamazem, 2002). Macropinocytosis normally starts with external stimuli as growth factors that lead to the activation of tyrosine kinase-like receptors, activation of a signaling cascade dependent of Rac1-Ras-related C3 botulinum toxin substrate, Pak1, PKC, Rab5, Arf6, and PI3K culminating in actin cytoskeleton remodeling and the formation of membrane ruffles (Mercer and Helenius, 2009). Inhibitors of Na^+^/H^+^ channel, such as amiloride and IPA, block membrane ruffling (Dowrick et al., 1993), making them a tool to study macropinocytosis (Dowrick et al., 1993). Macropinocytosis is also described as a process of internalization for *Legionella pneumophila* (Maréchal et al., 2001) and various viruses, such as vaccinia, adenovirus 3, herpes 1 and HIV (Mercer and Helenius, 2009), *Leishmania amazonensis* (Wanderley et al., 2006), and *T. cruzi* (Barrias et al., 2013).

In view of the existence of different endocytic processes, we decided to investigate whether any of them are involved in the internalization of *T. gondii* by different cell types. Our observations indicate that clathrin-mediated endocytosis and macropinocytosis are also important to the entry of this protozoan into the host cell.

## Materials and Methods

### Parasites and Cell Culture

*Toxoplasma gondii* tachyzoites from RH strain were maintained by passages in human foreskin fibroblast (HFF; kindly donated by Sheila Nardelli—ICC/FIOCRUZ-BR) cell culture. After 2–3 days of infection, the parasites obtained from the supernatant were centrifuged at 1,000 g for 10 min before use. The number of parasites in the supernatant was quantified in a Neubauer chamber.

Two types of host cells were used: mouse peritoneal macrophages and the HFF1 fibroblast cell line. The cells were cultivated in RPMI 1640 (Gibco) medium (peritoneal macrophages) or with high-glucose DMEM (HFF1) supplemented with 10% fetal bovine serum and maintained at 37°C in a 5% CO_2_ atmosphere. One day before the experiments, resident peritoneal macrophages were obtained by peritoneal washing of Swiss mice with Hank's solution, plated on glass coverslips and allowed to adhere for 1 h at 37°C in an atmosphere with 5% CO_2_. Then, the cells were washed with Hanks' solution wash, and RPMI 1640 medium with 10% FBS was added to the cells, which were cultured at 37°C in 5% CO_2_. The experimental protocol was approved by the Instituto de Biofisica Carlos Chagas Filho (Universidade Federal do Rio de Janeiro) Ethics Committee for Animal Experimentation.

### Host Cell-Parasite Interaction

Experiments were carried out with 3 × 10^3^ cells plated on 96-well flat clear-bottom black polystyrene TC-treated microplates (3904, Corning). The host cells were preincubated with 100 μM amiloride (A7419, Sigma-Aldrich), 5 μM IPA-3, 0,45 M sucrose (hypertonic medium), 10 μg/mL chlorpromazine, 20 nM Pitstop 2, 100 nM cytochalasin D, or 100 nM latrunculin for 1 h. Then, the cells were washed (three times) with phosphate-buffered saline (PBS), pH 7.2, and the parasites were added to the wells at a 10:1 parasite-host cell ratio. After 30 min of parasite interaction with the host cells, samples were taken and fixed with freshly prepared 4% formaldehyde in phosphate-buffered saline. The concentrations of the various drugs used were selected based on previous experiments carried out in the laboratory (Vieira et al., 2002; Barrias et al., 2012, 2013). After fixation, the cells were washed with PBS, incubated with permeabilized with 2% Triton X-100 in PBS for 10 min, incubated with the primary antibody from mouse hybridoma supernatant anti-SAG1 (1:1,000 dilution) (kindly gifted by Dr. Dominique Soldati, University of Geneva, Genève, Switzerland) diluted in blocking buffer for 30 min to label the parasites non-internalized. After these, the samples were incubated with 100 mM NH_4_Cl (30 min) and then incubated with PHEM buffer containing 3% bovine serum albumin (PHEM-BSA) for 30 min at room temperature. After these, the cells were incubated with Alexa Fluor 546-phalloidin (Molecular Probes) (1:40) to label the host cell actin cytoskeleton (used to separate intra and extracellular environment). After 45 min the cells were washed and incubated for 60 min with secondary goat anti-mouse antibody IgG Alexa Fluor 488 (1:800 dilution) (Molecular Probes). The cells were then incubated with the nuclear marker Hoechst 33342 (trihydrochloride, trihydrate, 100 mg—Thermo Fisher) (1:5,000). After images of all wells were acquired with an IN Cell Analyzer 2000 using a 20 × NA = 0.21 objective, segmentation was performed using the In Cell investigator program (module organelle analysis). The intracellular and extracellular parasites were separated by difference in label (intracellular tachyzoites appear with the nuclei label and the extracellular tachyzoite were label with Alexa 488). The separation between host cell and *T. gondii* was done through the difference of nucleus size. The parasite infection profile was evaluated by the indexes of adhesion and internalization. The adhesion index is obtained by multiplying the mean number of adhered *T. gondii* per host cell and the percentage of cells with attached parasites and the Internalization index is calculated by multiplying the mean number of internalized *T. gondii* per host cell and the percentage of infected cells. The data were plotted using GraphPad Prism 6.0 software. At least three independent experiments were carried out in duplicate. Statistical analysis was carried out using two-way ANOVA with Tukey's test. All values are presented as the means ± SD. The results were considered significant when *P* < 0.05.

#### Fluorescence Microscopy

For fluorescence microscopy, the host cells were plated as described above and incubated with *T. gondii*, as previously described for 15 min or 1 h. The cells were fixed in 4% formaldehyde in 0.1 M sodium phosphate buffer, pH 7.2, for 1 h, washed with PBS (pH 7.2) and permeabilized with a solution of 80% methanol and 20% acetone. Non-specific binding sites were blocked (3% bovine albumin serum, 0.025% Tween 20, and 0.25% fish gelatin in PBS, pH 8.0) for 1 h and incubated with an anti-PAK1 antibody (Life Technologies) (1:100) for 1 h. After this time, the cells were then washed with PBS (pH 7.2) and incubated with an Alexa Fluor 488-conjugated secondary antibody diluted in blocking buffer for 1 h. The nuclei were labeled with 4′,6′-diamidino-2-phenylindole (DAPI) (1 μg/mL, Sigma) for 5 min. Coverslips were mounted onto the slides using prolonged antifade (Molecular Probes). Observations were made using a CLSM Leica TCS SP5 microscope.

#### Transmission Electron Microscopy

In order to analyze the ultrastructure of the cells, the samples in culture flasks were treated with the inhibitors of the entry mechanisms described and infected with *T. gondii*, as described above. After 1 h, the cells were fixed with 2.5% glutaraldehyde in 0.1 M sodium cacodylate buffer, pH 7.4. The cells were scraped off the flasks with a rubber policeman, washed with 0.1 M sodium cacodylate buffer and post-fixed for 1 h in the dark with 1% osmium tetroxide in 0.1 M sodium cacodylate buffer. The cells were washed, dehydrated in acetone, and embedded in Epon. Ultrathin sections were stained using uranyl acetate and lead citrate and then analyzed under a FEI Tecnai Spirit transmission electron microscope at the National Center for Structural Biology and Bioimaging (CENABIO) multiuser unit of UFRJ.

#### Scanning Electron Microscopy

For scanning electron microscopy, the samples were treated with inhibitors and infected with *T. gondii*, as described above; fixed for 1 h in a solution containing 2.5% glutaraldehyde in 0.1 M sodium cacodylate buffer, pH 7.4; and post-fixed for 1 h in the dark with a solution containing 1% osmium tetroxide in 0.1 M. The cells were washed, dehydrated in acetone, critical point-dried, and mounted on stubs. The samples were coated with platinum (5 nm) and observed using an Auriga 40 scanning electron microscope at the National Center for Structural Biology and Bioimaging (CENABIO) multiuser unit of UFRJ.

## Results

### Inhibition of Clathrin-Coated Pit Formation Impairs *T. gondii* Internalization

**Figure 1:**
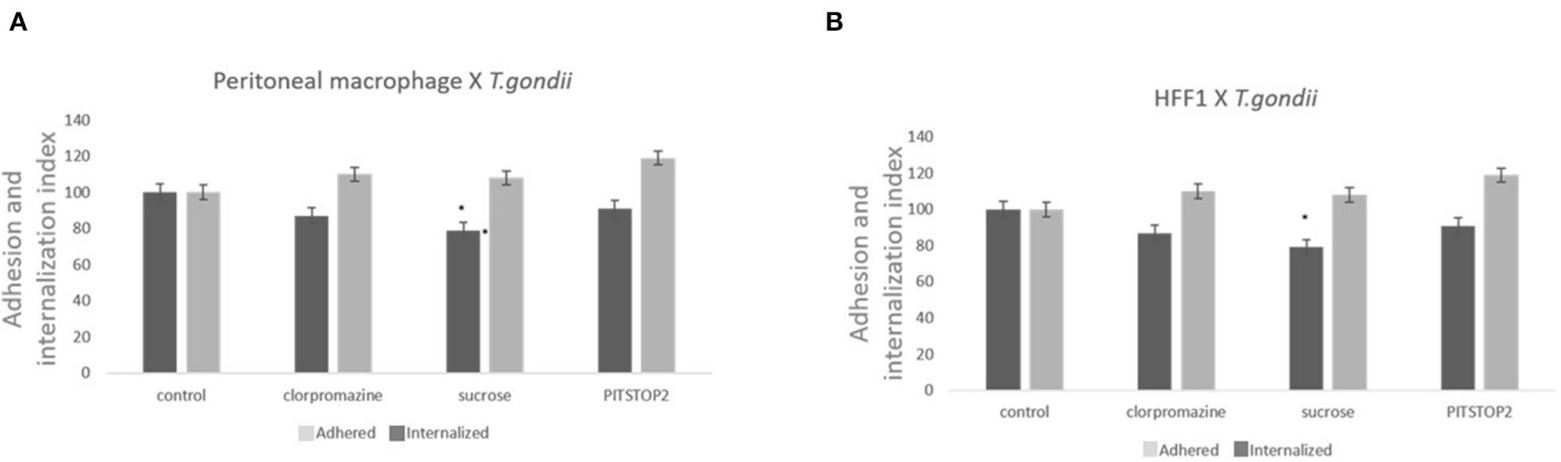
Inhibitors against the clathrin pathway promote the inhibition of *T. gondii* internalization. Macrophages **(A)** and HFF1 cells **(B)** treated with chlorpromazine hydrochloride (10 μg/mL), sucrose hypertonic medium (0.45 M), and pitstop 2 (20 nM), for 60 min, washed with PBS (three times) and then infected with *T. gondii* (30 min), as described in the Materials and Methods. Note that after all treatments there is a significant reduction of entry into both cell types when compared with control (* indicates significant difference in relation to the control with value of *p* < 0.05).

Three classical inhibitors of clathrin-mediated endocytic processes—chlorpromazine, sucrose hypertonic medium, and pitstop 2- were used to analyze the participation of clathrin-coated pits in *T. gondii* entry into host cells. To avoid the formation of clathrin-coated pits, peritoneal macrophages and HFF1 cells were treated with chlorpromazine hydrochloride (10 μg/mL), sucrose hypertonic medium (0.45 M), or pitstop 2 (20 μM) and allowed to interact with *T. gondii*. The internalization rate of *T. gondii* in the peritoneal macrophages treated with chlorpromazine, sucrose and pitstop 2 was reduced by ~30% compared to the rate found for the untreated cells (Figure 1A). A less prominent inhibition (about 20% inhibition—Figure 1B) was observed when HFF1 fibroblasts were used as host cell model. In both instances, the reduction in internalization was accompanied by an increase of 10% in the adhesion index of parasites to the host cell surface in comparison with the control.

### Amiloride and IPA-3 Significantly Block *T. gondii* Invasion Into Host Cells

**Figure 2:**
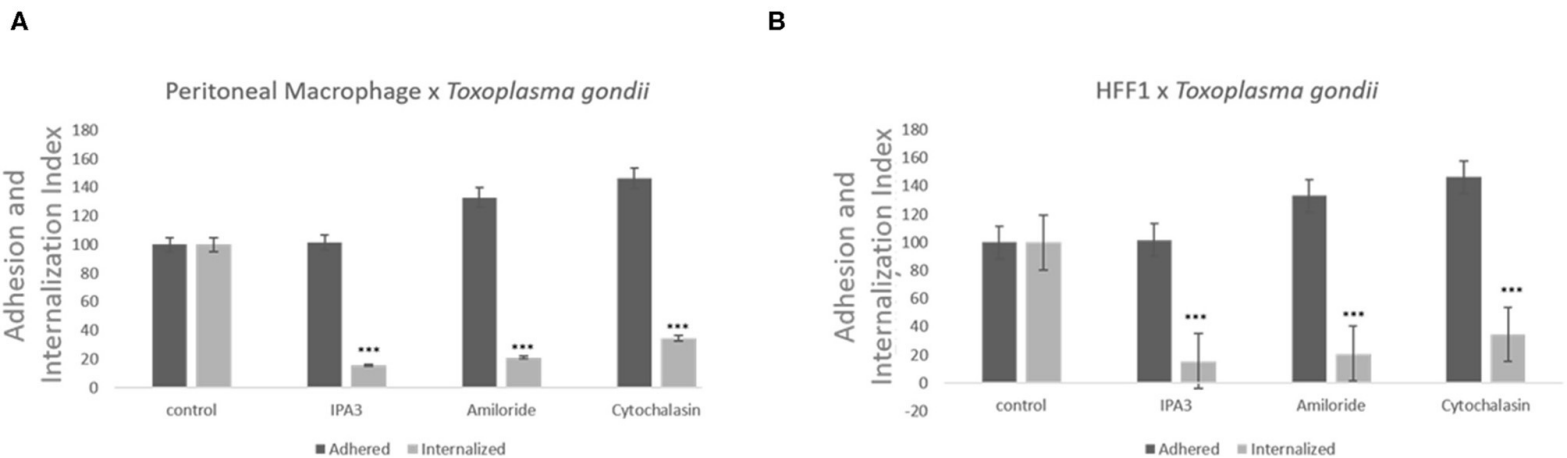
Inhibitors against the macropinocytosis pathway, such as those against Na^+^/H^+^ exchangers and PAK1, promote a significant decrease in *T. gondii* invasion. Macrophages **(A)** and HFF1 cells **(B)** previously treated with IPA3 (5 μM), amiloride (100 μM), and cytochalasin D **(**100 nM), for 60 min, washed with PBS (three times) and then infected with *T. gondii* (30 min), as described in the Materials and Methods. Note that after all treatments there is a significant reduction of entry into both cell types when compared with control (*** indicates significant difference in relation to the control with value of *p* < 0.05).

Treatment with of the inhibitor amiloride for 1 h was used to determine whether macropinocytosis is involved in *T. gondii* entry into host cells, peritoneal macrophages and HFF1. After treatment, and before the parasites were added, the medium with amiloride was removed in order to prevent and ensure that the inhibitor was only acting on host cells and not on the parasites. As shown in Figure 2, at all concentrations tested, amiloride significantly interfered with internalization by both cell types. In the case of HFF1 cells, the reduction in internalization was of ~60%, while for peritoneal macrophages, the reduction was of 80% (Figures 2A,B). In relation to parasite adhesion, in both cell types, we observed increases of 30 and 40%, respectively (Figures 2A,B). To test reversibility of the inhibition, the peritoneal macrophages were treated with 100 μM amiloride for 1 h, washed and then incubated for 120 min with RPMI medium before the addition of parasites. We observed that both the adhesion and internalization indexes were similar to those observed for the untreated cells, indicating that the effect of amiloride was completely reversible, with internalization showing the same pattern of interaction as the control without treatment (not shown). Treatment with IPA-3 was performed in the same manner as the with amiloride treatment, at a 5 μM concentration. This treatment significantly affected the adhesion and the internalization of the parasites in both types of cells (Figures 2A,B). The values of adhesion and internalization obtained for the treatment with IPA3 were the same as those observed for the treatment with amiloride.

### Inhibitors of Endocytic Pathways Block the Formation of Parasitophorous Vacuoles

**Figure 3:**
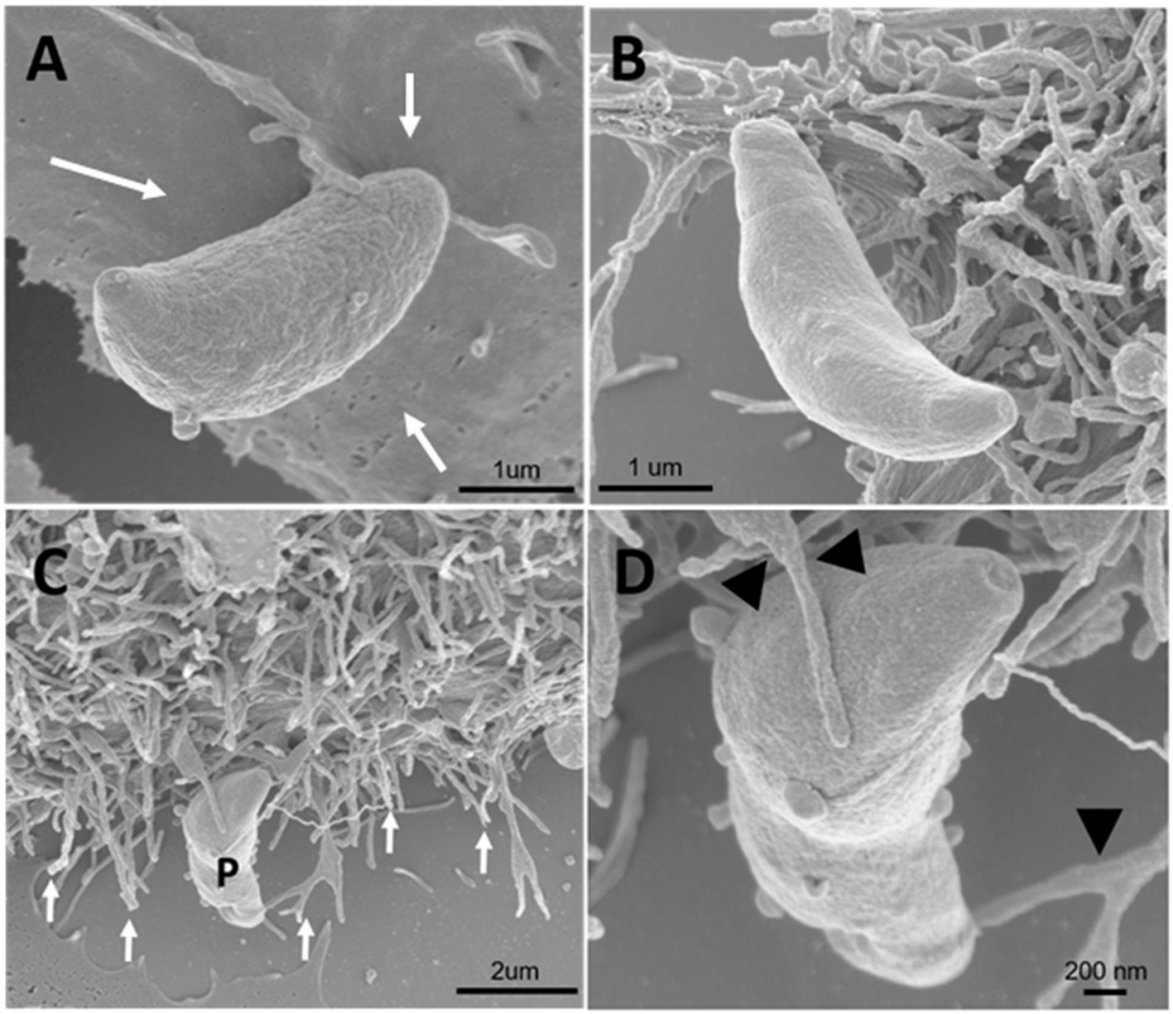
Field emission scanning electron microscopy of the HFF1 cells treated with amiloride or sucrose hypertonic medium, washed with PBS (three times) and allowed to interact with tachyzoites. **(A–D)** After 30 min of interaction with the cells the parasites attached to the membrane of the host cells (white arrows). **(B–D)** After 30 min of parasite interaction with the cells that were previously treated with hypertonic medium of sucrose, a large amount of membrane projections was observed around the parasite attached to the cell (arrows and arrowheads).

**Figure 4:**
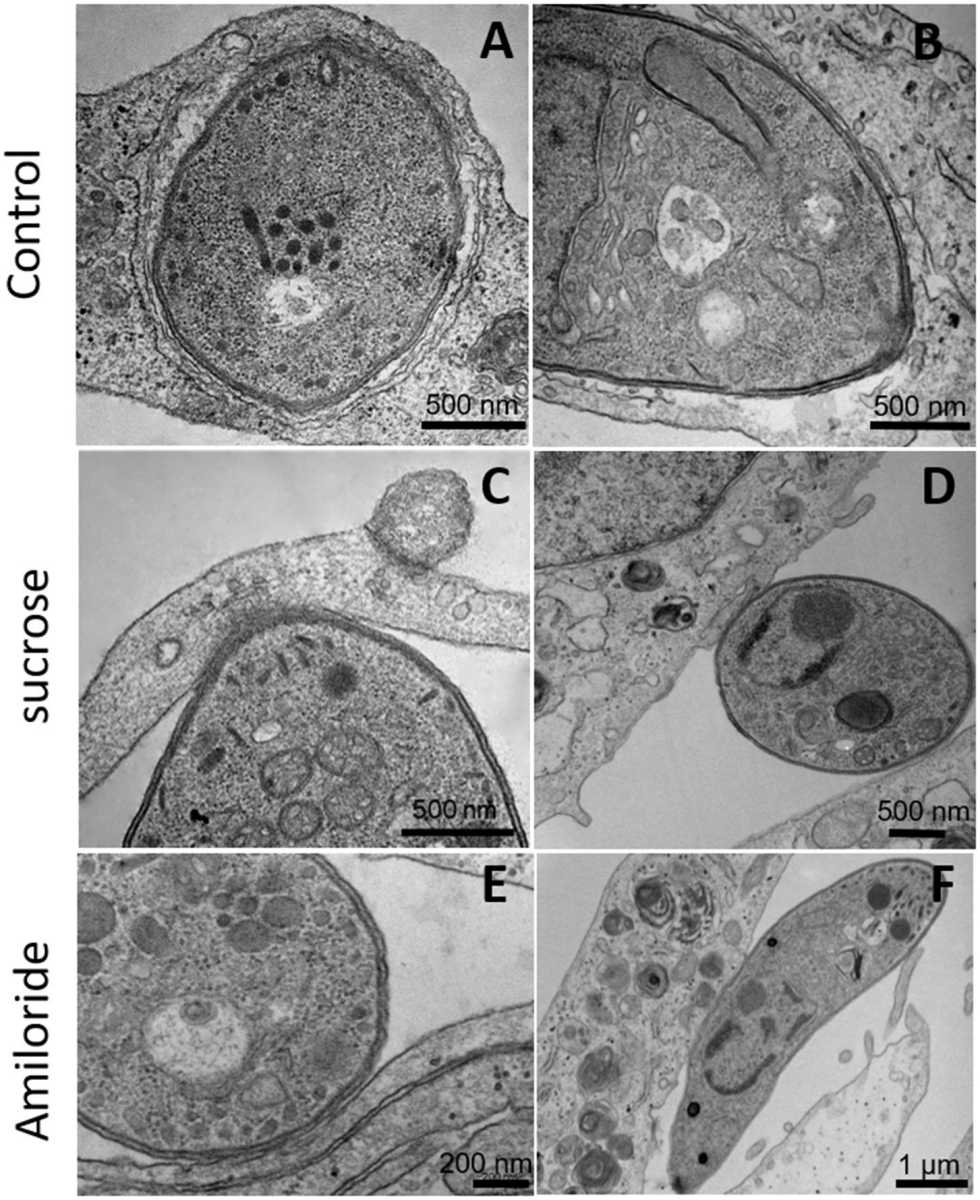
Transmission electron microscopy of HFF1 treated with amiloride or sucrose hypertonic medium, washed with PBS (three times) and allowed to interact with tachyzoites for 1 h. **(A,B)** HFF cells that were untreated had *T. gondii* inside the parasitophorous vacuole. **(C–F)** HFF cells that were previously treated with sucrose or amiloride were found with most of the parasites attached to their surface.

Since inhibitors of macropinocytosis and the clathrin-mediated endocytosis pathway were able to inhibit the entry of *T. gondii* tachyzoites, we decided to investigate the *T. gondii* entry profile. Scanning electron microscopy showed that, after 30 min of interaction (enough time for the protozoan to fully enter host cells), tachyzoites still were remained attached to treated fibroblasts (Figures 3A–D). The treatment with the clathrin inhibitor was the only treatment shown to significantly inhibit parasite entry into fibroblasts and we found that, in addition to parasites merely remaining adhered to the host cell plasma membrane, the host cells exhibited a large number of nanotubule-like plasma membrane projections where the parasite appears to be attached (Figures 3B–D). Note that although they are not completely internalized, they appear partially recovered with the plasma membrane of the host cells with projections similar to philopodia or nanotubules (arrowheads and arrows—Figures 3C,D). These, in turn, appear in large quantities in HFF1 after treatment, while no such structures are seen in untreated cells (white arrows—Figure 3A). Transmission electron microscopy corroborated the findings obtained by scanning microscopy, showing that parasites merely adhered to the host cell membrane after 30 min of interaction after the treatments (Figures 4B–F), while for the untreated cells, the parasites were fully internalized at the time of interaction (Figures 4A,B).

### Rabankyrin 5 and Pak1 Co-localize With Parasitophorous Vacuoles

**Figure 5:**
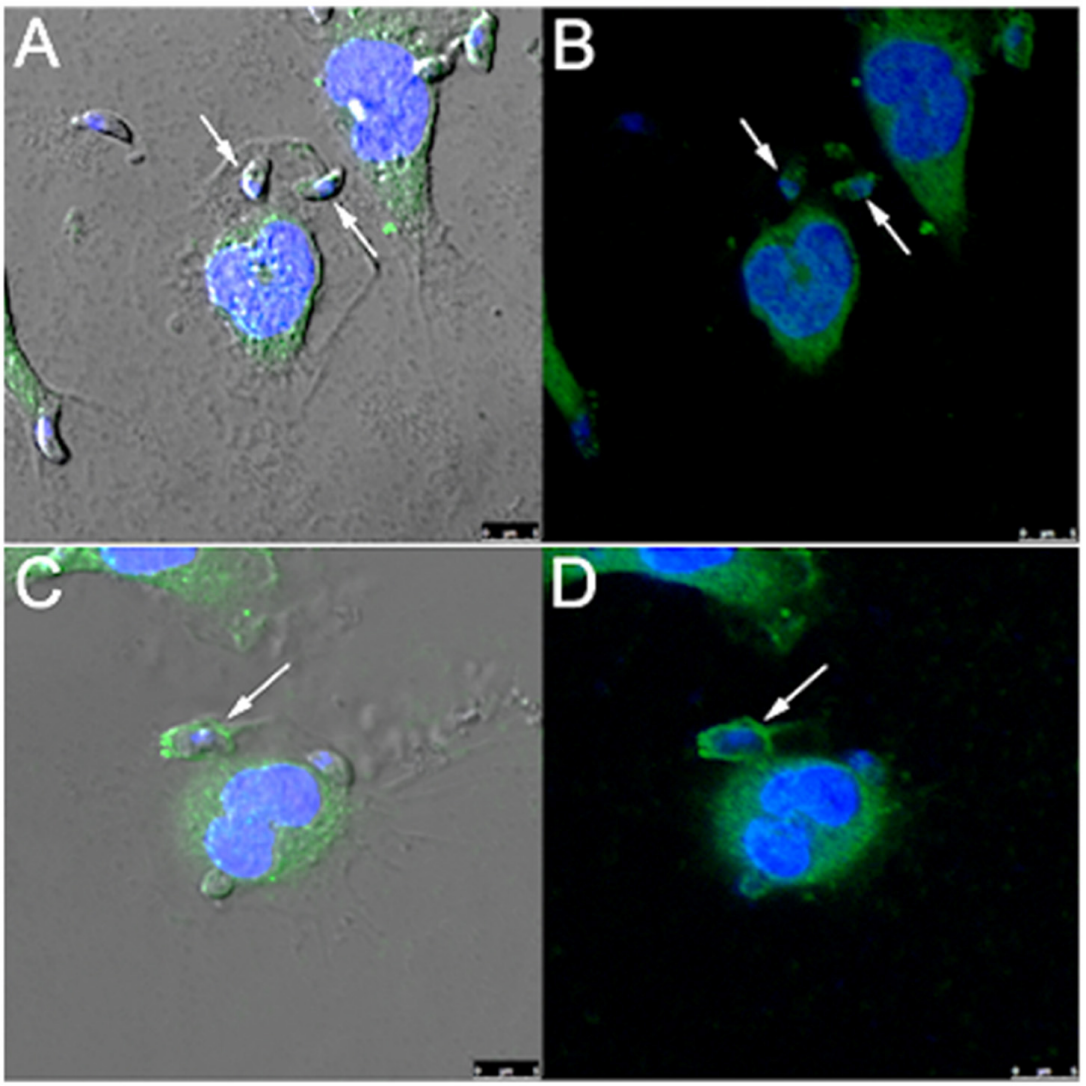
*T. gondii* co-localizes with rabankyrin 5 and PAK1 into the parasitophorous vacuole in peritoneal macrophages. Tachyzoites were incubated with peritoneal macrophages for 1 h. After that, cells were fixed and processed for observation by confocal laser scanning microscopy. **(A,B)** DAPI (blue), anti-rabankyrin 5 Alexa 488 (green); **(C,D)** DAPI (blue), anti Pak1 Alexa 488 (green). Note that tachyzoites (arrow) co-localize with rabankyrin 5 and Pak1.

Since rabankyrin 5 protein (effector of Rab5) is associated with macropinosomes it could be considered a molecular marker for macropinocytosis. Furthermore Pak1 is activated by the small GTPase Rac1 (Ras-related C3 botulinum toxin substrate) and is related to macropinocytosis of Ad3, vaccinia virus and *T. cruzi* (Al Soraj et al., 2012; Barrias et al., 2013; Sánchez et al., 2017). To check if macropinocytosis can be a path for *T. gondii* entry into host cells, we used anti-rabankyrin 5 and anti-Pak1 antibodies to label areas described as macropinosomes. We observed that both antibodies labeled the site of entry of some, but not all, parasites, as well as the formed parasitophorous vacuole in the macrophages (Figure 5).

## Discussion

As an obligatory intracellular parasite, *T. gondii* have developed strategies to gain access and enter host cells. Studies on the mechanisms utilized by *T. gondii* to reach this goal, key for its survival and multiplication, are necessary not only to fully understand the biology of the parasite, but is also important for the identification of novel drug targets for the treatment of toxoplasmosis. The involvement of different kinases (Coppens et al., 2006), the participation of actin filaments (Silva et al., 1982), and the involvement of cholesterol, planar lipid rafts, and caveolae (Coppens and Joiner, 2003) in the internalization process are well-established. Nevertheless, it is also known that, in contrast to *T. cruzi* (Barrias et al., 2013) and *Leishmania* (De Souza et al., 2010; Kumar et al., 2019), also intracellular parasites, organelles of the endocytic pathway do not fuse with the *T. gondii* parasitophorous vacuole (Sinai, 2008); *T. gondii* uses a non-canonical autophagy pathway to recruits LC3b to the PV and retains organelles in the endocytic pathways, such as late endosomes and lysosomes, near the PV to access cell nutrients (reviewed by Coppens et al., 2006).

It has been assumed that a considerable amount of the *T. gondii* population, actively invade the host cells by a mechanism conducted by the own parasite, named active invasion. In this process, the parasite secretes proteins from organelles like micronemes and rhoptries which will mediate the invasion (Carruthers and Sibley, 1997). This mechanism includes the assembly of a transitory structure named mobile junction, composed by parasites and host cell's proteins forming a complex beneath host cell PM (reviewed by Horta et al., 2020). Rhoptry proteins (RONs) form a molecular complex with host cell proteins of the ESCRT family. This connects the parasite to the host cell actin cytoskeleton (Guérin et al., 2017), while the extracellular domain of a specific rhoptry protein RON2, that is on the host cell cytoplasm, binds to AMA1, a protein secreted by micronemes, present on parasite plasma membrane. Inside the parasite, the cytosolic domain of AMA1 binds to a parasite actin-myosin motor to allow parasite moving toward the parasitophorous vacuole in process of formation (reviewed by Horta et al., 2020). This junction allows the parasite entry and the formation of a parasitophorous vacuole that is protective to the parasite because its composition exclude proteins from the host endocytic pathways.

A number of mechanisms have been proposed for internalization of *T. gondii* in host cells (Morisaki et al., 1995; Walker et al., 2014). Jones et al. (1972) have shown the participation of the host cells in the entry process and the intracellular fate of *Toxoplasma* in macrophages, HeLa cells and fibroblasts. The mechanism of entry into these cells was described as phagocytosis, with the presence of pseudopods extended by the cells around the parasites with subsequent formation of a typical phagocytic vacuole. Another interesting feature observed in the *Toxoplasma* infection in macrophages was the ability of this cell to control the parasite growth in relation to the HeLa and fibroblasts (Jones and Hirsch, 1972).

Macropinocytosis differs from other types of endocytosis for its unique susceptibility to inhibitors of Na^+^/H^+^ exchange transporters that regulate cytoplasmic acidification (Koivusalo et al., 2010). Our present observations indicate that treatment of macrophages with amiloride, an inhibitor of macropinocytosis, significantly inhibited the internalization of *T. gondii*, suggesting that macropinocytic machinery play some role on parasite internalization. We observed that, in contrast to the internalization index, the adhesion index was not significantly reduced in macrophages, an indication that the receptors in the host cell plasma membrane were not affected. Previously, Koivusalo et al. (2010) demonstrated that cytoplasmic acidification did not alter receptor engagement or phosphorylation and did not significantly depressed phosphatidylinositol-3-kinase activation in cells treated with amiloride. On the other hand, the remodeling of the cytoskeleton, characteristic of macropinocytosis, requires activity of phosphatidylinositol-3-kinase (PI3K) at the plasma membrane (Araki et al., 1996; Rupper et al., 2001; Lindmo and Stenmark, 2006). Actin remodeling resulting from activation of GTPases, such as Rac1 and Cdc42, was found to be extremely sensitive to submembranous pH. In consequence of this requirement, Na^+^/H^+^ exchange is essential to promote actin polymerization during macropinocytosis and changes in pH resulting from amiloride treatment would significantly alter the signaling and cytoskeleton rearrangements typical of macropinocytosis. Other studies have shown that *T. gondii*-induced activation of host cell PI3-kinase and positively correlates with the efficient invasion of non-professional phagocytic cells and macrophages (Da Silva et al., 2009; Zhou et al., 2013), suggesting the participation of actin microfilaments in parasite invasion and supporting the idea that the macropinocytic pathway takes part in this process. Indeed, in previous studies, when host cells were treated with cytochalasin D, a drug that interferes with actin filaments, a significant inhibition of the internalization of *T. gondii* by host cells occurred (Silva et al., 1982; Dobrowolski and Sibley, 1996). Although macropinocytosis inhibitors inhibited up to 80% the internalization rate of *Toxoplasma*, we do not want to infer that only the remaining 20% of parasites enter the cell through the classical active entry mechanism. We cannot exclude the possibility that drug treatments that block macropinocytosis also interfere with steps of the active process. As the treatment with the inhibitors disturbs the plasma membrane charges or even the actin microfilaments array, it may also affect the *T. gondii* active entry, or any other mechanism involving rearrangement of actin and specific membrane charge/receptors, besides macropinocytosis. We even cannot exclude the possibility that active penetration and macropinocytosis are complementary mechanisms, since one of them is directed by the parasite and the other by the host cell. Further studies are necessary to clarify this point.

It is important to point out that treatment of the cells with the various inhibitors of macropinocytosis and clathrin-mediated endocytic activity does not interfere with cell viability. In addition, the cells are incubated in the presence of the various inhibitors and washed before the addition of the parasites, so they do not interfere with any parasite structure.

Morphological analysis of the entry process in mice peritoneal macrophages, that had been incubated with amiloride for 1 h the internalization of *T. gondii* was prevented. At this time point, parasite bodies were partially surrounded by host cell plasma membrane projections. Similar results were obtained by treating peritoneal macrophages with the PI-3 kinase inhibitors wortmannin and LY294002 and dynasore, an inhibitor of dynamin (Caldas et al., 2009, 2013). However, a close contact between the host cell plasma membrane and the parasite was observed in these studies, while in macrophages treated with amiloride, we observed projections of the plasma membrane loosely surrounding the parasite. The lack of contact between the host cell membrane and the parasite was expected, as it is a feature of the initial macropinocytic process.

Schnatwinkel et al. (2004) described a novel PI (3)P-binding Rab5 effector, designated rabankyrin-5, that localizes in large vacuolar structures that correspond to macropinosomes in epithelial cells and fibroblasts. Overexpression of rabankyrin-5 increases the number of macropinosomes and stimulates fluid-phase uptake, whereas its downregulation inhibits these processes. These observations pointed to rabankyrin 5 as a possible marker for macropinosomes. Here, we observed that rabankyrin 5 protein is located at *T. gondii* entry sites, as well as in some parasitophorous vacuoles found in macrophages. It is important to note that rabankyrin 5 is incorporated into macropinosomes immediately after their formation. However, in the case of vacuoles containing *T. gondii*, the situation is more complex because, as is well-known, the parasite blocks all fusion of the PVs with components of the endocytic pathway (Joiner et al., 1990). Here, we observed that macropinocytosis inhibitors can cause a drastic reduction in tachyzoites internalization. In another study, our group demonstrated that lipid rafts are an important pathway for *T. gondii* invasion of host cells (Cruz et al., 2014).

Clathrin-mediated endocytosis has been reported as important to the internalization of different particles and microorganism (including pathogenic bacteria, fungi, and viruses) into host cells (Humphries and Way, 2013; Słońska et al., 2016). These findings contradict the classic model of coated pit formation where, due to the stereological features of the triskelion, the maximum size of clathrin vesicles is ~150 nm (Humphries and Way, 2013). The internalization of pathogens with a diameter >1 μm via clathrin-pathway is currently considered an exception that is inconsistent with the classical view. Here, we observed that incubation of the cells with inhibitors of the assembly of clathrin-coated pits (chlorpromazine and hypertonic sucrose medium) (Anderson et al., 1982; Robertson et al., 2014) inhibited the internalization of *T. gondii*, although a drastic reduction was not detected. However, inhibition was more pronounced in the HFF1 cells than in the macrophages. Chlorpromazine is also described as a molecule that interferes with the biogenesis of large intracellular vesicles as phagosomes and macropinosomes (Elferink, 1979; Watarai et al., 2001). The same was described to hypertonic sucrose, that presents the ability to decrease the number of both clathrin-coated pits as well as to reduce macropinocytosis and lipid rafts.

Taken together, our results reinforce an important and the active role of the host cell in the process of interaction of *T. gondii*-host cell that results in parasite entry. Usually, more emphasis has been given to the active role of the parasite in the invasion process. This is based on the observation of successive secretory events associated with by protozoan motility activation, extrusion of the conoid, assembly of the “moving junction,” and formation of the parasitophorous vacuole. Our results indicate that other endocytic processes controlled by the host cell, such as macropinocytosis and chlatrin-mediated endocytosis, also play a role for the success interplay between the host and the parasite.

## Data Availability Statement

All datasets generated for this study are included in the article/supplementary material.

## Author Contributions

All authors contributed to manuscript revision, read, and approved the submitted version.

## Conflict of Interest

The authors declare that the research was conducted in the absence of any commercial or financial relationships that could be construed as a potential conflict of interest.

## References

[B1] Al Soraj M. He L. Peynshaert K. Cousaert J. Vercauteren D. Braeckmans K.. (2012). siRNA and pharmacological inhibition of endocytic pathways to characterize the differential role of macropinocytosis and the actin cytoskeleton on cellular uptake of dextran and cationic cell penetrating peptides octaarginine (R8) and HIV-Tat. J. Control. Release 161, 132–141. 10.1016/j.jconrel.2012.03.015 22465675

[B2] Anderson R. G. Brown M. S. Beisiegel U. Goldstein J. L. (1982). Surface distribution and recycling of the low density lipoprotein receptor as visualized with antireceptor antibodies. J. Cell Biol. 93, 523–531. 10.1083/jcb.93.3.523 6288727 PMC2112164

[B3] Andersson E. R. (2012). The role of endocytosis in activating and regulating signal transduction. Cell. Mol. Life Sci. 69, 1755–1771. 10.1007/s00018-011-0877-1 22113372 PMC11114983

[B4] Araki N. Johnson M. T. Swanson J. A. (1996). A role for phosphoinositide 3-kinase in the completion of macropinocytosis and phagocytosis by macrophages. J. Cell. Biol. 135, 1249–1260. 10.1083/jcb.135.5.1249 8947549 PMC2121091

[B5] Barrias E. S. de Carvalho T. M. De Souza W. (2013). *Trypanosoma cruzi*: entry into mammalian host cells and parasitophorous vacuole formation. Front. Immunol. 4:186. 10.3389/fimmu.2013.00186 23914186 PMC3730053

[B6] Barrias E. S. Reignault L. C. De Souza W. Carvalho T. M. (2012). *Trypanosoma cruzi* uses macropinocytosis as an additional entry pathway into mammalian host cell. Microbes Infect. 14, 1340–1351. 10.1016/j.micinf.2012.08.003 23010292

[B7] Blader I. J. Saeij J. P. (2009). Communication between Toxoplasma gondii and its host: impact on parasite growth, development, immune evasion, and virulence. APMIS 117, 458–476. 10.1111/j.1600-0463.2009.02453.x 19400868 PMC2810527

[B8] Blanchard E. Belouzard S. Goueslain L. Wakita T. Dubuisson J. Wychowski C.. (2006). Hepatitis C virus entry depends on clathrin-mediated endocytosis. J. Virol. 80, 6964–6972. 10.1128/JVI.00024-06 16809302 PMC1489042

[B9] Breyner N. M. Hecht M. Nitz N. Rose E. Carvalho J. L. (2020). *In vitro* models for investigation of the host-parasite interface - possible applications in acute chagas disease. Acta Trop. 202:105262. 10.1016/j.actatropica.2019.105262 31706861

[B10] Caldas L. A. Attias M. De Souza W. (2009). Dynamin inhibitor impairs Toxoplasma gondii invasion. FEMS Microbiol. Lett. 301, 103–108. 10.1111/j.1574-6968.2009.01799.x 19817867

[B11] Caldas L. A. Seabra S. H. Attias M. De Souza W. (2013). The effect of kinase, actin, myosin and dynamin inhibitors on host cell egress by Toxoplasma gondii. Parasitol. Int. 62, 475–482. 10.1016/j.parint.2013.04.006 23624149

[B12] Carruthers V. Boothroyd J. C. (2007). Pulling together: an integrated model of toxoplasma cell invasion. Curr. Opin. Microbiol. 10, 83–89. 10.1016/j.mib.2006.06.017 16837236

[B13] Carruthers V. B. Giddings O. K. Sibley L. D. (1999). Secretion of micronemal proteins is associated with Toxoplasma invasion of host cells. Cell. Microbiol. 1, 225–235. 10.1046/j.1462-5822.1999.00023.x 11207555

[B14] Carruthers V. B. Håkansson S. Giddings O. K. Sibley L. D. (2000). Toxoplasma gondii uses sulfated proteoglycans for substrate and host cell attachment. Infect Immun. 68, 4005–4011. 10.1128/iai.68.7.4005-4011.2000 10858215 PMC101681

[B15] Carruthers V. B. Sibley L. D. (1997). Sequential protein secretion from three distinct organelles of Toxoplasma gondii accompanies invasion of human fibroblasts. Eur. J. Cell Biol. 73, 114–123. 9208224 PMC13475723

[B16] Cooper A. Shaul Y. (2006). Clathrin-mediated Endocytosis and Lysosomal Cleavage of Hepatitis B Virus Capsid-Like Core Particles. J. Biol. Chem. 281, 16563–16569. 10.1074/jbc.M601418200 16618702

[B17] Coppens I. Dunn J. D. Romano J. D. Pypaert M. Zhang H. Boothroyd J. C.. (2006). *Toxoplasma gondii* sequesters lysosomes from mammalian hosts in the vacuolar space. Cell. 125, 261–274. 10.1016/j.cell.2006.01.056 16630815

[B18] Coppens I. Joiner K. A. (2003). Host but not parasite cholesterol controls Toxoplasma cell entry by modulating organelle discharge. Mol. Biol. Cell 14, 3804–3820. 10.1091/mbc.e02-12-0830 12972565 PMC196568

[B19] Cruz K. D. Cruz T. A. Veras de Moraes G. Paredes-Santos T. C. Attias M. de Souza W. (2014). Disruption of lipid rafts interferes with the interaction of Toxoplasma gondii with macrophages and epithelial cells. Biomed. Res. Int. 2014:687835. 10.1155/2014/687835 24734239 PMC3964738

[B20] Da Silva C. V. Da Silva E. A. Cruz M. C. Chavrier P. Mortara R. A. (2009). ARF6, PI3-kinase and host cell actin cytoskeleton in Toxoplasma gondii cell invasion. Biochem Biophys Res Commun. 378, 656–661. 10.1016/j.bbrc.2008.11.108 19061866

[B21] Daher W. Soldati-Favre D. (2009). Mechanisms controlling glideosome function in apicomplexans. Curr. Opin. Microbiol. 12, 408–414. 10.1016/j.mib.2009.06.008 19577950

[B22] De Souza W. De Carvalho T. M. (2013). Active penetration of Trypanosoma cruzi into host cells: historical considerations and current concepts. Front. Immunol. 4:2. 10.3389/fimmu.2013.00002 23355838 PMC3555119

[B23] De Souza W. de Carvalho T. M. Barrias E. S. (2010). Review on *Trypanosoma cruzi*: host cell interaction. Int. J. Cell. Biol. 2010:295394. 10.1155/2010/295394 20811486 PMC2926652

[B24] Dobrowolski J. M. Sibley L. D. (1996). Toxoplasma invasion of mammalian cells is powered by the actin cytoskeleton of the parasite. Cell. 84, 933–939. 10.1016/s0092-8674(00)81071-5. 8601316

[B25] Doherty G. J. McMahon H. T. (2009). Mechanisms of endocytosis. Annu. Rev. Biochem. 78, 857–902. 10.1146/annurev.biochem.78.081307.110540 19317650

[B26] Dowrick P. Kenworthy P. McCann B. Warn R. (1993). Circular ruffle formation and closure lead to macropinocytosis in hepatocyte growth factor/scatter factor-treated cells. Eur. J. Cell. Biol. 61, 44–53. 8223707

[B27] Dubremetz J. F. Achbarou A. Bermudes D. Joiner K. A. (1993). Kinetics and pattern of organelle exocytosis during *Toxoplasma gondii*/host-cell interaction. Parasitol. Res. 79, 402–408. 10.1007/bf00931830 8415546

[B28] Elferink J. G. (1979). Chlorpromazine inhibits phagocytosis and exocytosis in rabbit polymorphonuclear leukocytes. Biochem. Pharmacol. 28, 965–968. 10.1016/0006-2952(79)90287-9 375938

[B29] Feng M. Zhang J. Xu W. Wang H. Kong X. Wu X. (2018). Bombyx mori nucleopolyhedrovirus utilizes a clathrin and dynamin dependent endocytosis entry pathway into BmN cells. Virus Res. 253, 12–19. 10.1016/j.virusres.2018.05.020 29807041

[B30] Frénal K. Dubremetz J. F. Lebrun M. Soldati-Favre D. (2017). Gliding motility powers invasion and egress in apicomplexa. Nat. Rev. Microbiol. 15, 645–660. 10.1038/nrmicro.2017.86 28867819

[B31] Guérin A. Corrales R. M. Parker M. L. Lamarque M. H. Jacot D. El Hajj H.. (2017). Efficient invasion by *Toxoplasma* depends on the subversion of host protein networks. Nat. Microbiol. 2, 1358–1366. 10.1038/s41564-017-0018-1 28848228

[B32] Haas T. A. Plow E. F. (1994). Integrin-ligand interactions: a year in review. Curr. Opin. Cell Biol. 6, 656–662. 10.1016/0955-0674(94)90091-4 7833046

[B33] Horta M. F. Andrade L. O. Martins-Duarte É. S. Castro-Gomes T. (2020). Cell invasion by intracellular parasites - the many roads to infection. J. Cell Sci. 133:jcs232488. 10.1242/jcs.232488 32079731

[B34] Huang H. C. Chen C. C. Chang W. C. Tao M. H. Huang C. (2012). Entry of hepatitis B virus into immortalized human primary hepatocytes by clathrin-dependent endocytosis. J. Virol. 86, 9443–9453. 10.1128/JVI.00873-12 22740403 PMC3416113

[B35] Humphries A. C. Way M. (2013). The non-canonical roles of clathrin and actin in pathogen internalization, egress and spread. Nat. Rev. Microbiol. 11, 551–560. 10.1038/nrmicro3072 24020073

[B36] Johannes L. Lamazem C. (2002). Clathrin-dependent or not: is it still the question? Traffic 3, 443–451. 10.1034/j.1600-0854.2002.30701.x 12047552

[B37] Joiner K. A. Furhman S. A. Miettinen H. M. Kasper L. H. Mellman I. (1990). *Toxoplasma gondii*: fusion competence of parasitophorous vacuoles in Fc receptor-transfected fibroblasts. Science 249, 641–646. 10.1126/science.2200126 2200126

[B38] Jones T. C. Hirsch J. G. (1972). The interaction between toxoplasma gondii and mammalian cells: II. The absence of lysosomal fusion with phagocytic vacuoles containing living parasites. J. Exp. Med. 136, 1173–1194. 10.1084/jem.136.5.1173 4343243 PMC2139290

[B39] Jones T. C. Yeh S. Hirsch J. G. (1972). The interaction between toxoplasma gondii and mammalian cells: I. Mechanism of entry and intracellular fate of the parasite. J. Exp. Med. 136, 1157–1172. 10.1084/jem.136.5.1157 PMC2139313 5082671

[B40] Kanjee U. Rangel G. W. Clark M. A. Duraisingh M. T. (2018). Molecular and cellular interactions defining the tropism of *Plasmodium vivax* for reticulocytes. Curr. Opin. Microbiol. 46, 109–115. 10.1016/j.mib.2018.10.002 30366310 PMC6688184

[B41] Koivusalo M. Welch C. Hayashi C. Scott C. C. Kim M. (2010). Amiloride ihibits macropinocytosis by lowering submembranous pH and preventing Rac1 and Cdc42 signaling. J. Cell. Biol. 188, 547–563. 10.1083/jcb.200908086 20156964 PMC2828922

[B42] Kumar G. A. Karmakar J. Mandal C. Chattopadhyay A. (2019). Leishmania donovani internalizes into host cells via caveolin-mediated endocytosis. Sci. Rep. 9:12636. 10.1038/s41598-019-49007-1 31477757 PMC6718660

[B43] Latomanski E. A. Newton H. J. (2019). Taming the triskelion: bacterial manipulation of clathrin. Microbiol. Mol. Biol. Rev. 83:e00058–18. 10.1128/MMBR.00058-18 30814130 PMC6684001

[B44] Lidani K. C. F. Bavia L. Ambrosio A. R. de Messias-Reason I. J. (2017). The complement system: a prey of Trypanosoma cruzi. Front. Microbiol. 8:607. 10.3389/fmicb.2017.00607 28473804 PMC5397499

[B45] Lindmo K. Stenmark H. (2006). Regulation of membrane traffic by phosphoinositide 3-kinases. J. Cell. Sci. 119(Pt 4), 605–614. 10.1242/jcs.02855 16467569

[B46] Maréchal V. Prevost M. C. Petit C. Perret E. Heard J. M. Schwartz O. (2001). Human immunodeficiency virus type 1 entry into macrophages mediated by macropinocytosis. J. Virol. 75, 11166–11177. 10.1128/JVI.75.22.11166-11177.2001 11602756 PMC114696

[B47] Marsh M. Helenius A. (2006). Virus entry: open sesame. Cell 124, 729–740. 10.1016/j.cell.2006.02.007 16497584 PMC7112260

[B48] Mayor S. Pagano R. E. (2007). Pathways of clathrin-independent endocytosis. Nat. Rev. Mol. Cell. Biol. 8, 603–612. 10.1038/nrm2216 17609668 PMC7617177

[B49] Mayor S. Parton R. Donaldson J. G. (2014). Clathrin-independent pathways of endocytosis. Cold Spring Harb Perspect Biol. 6:a016758. 10.1101/cshperspect.a016758 24890511 PMC4031960

[B50] Mercer J. Helenius A. (2009). Virus entry by macropinocytosis. Nat. Cell. Biol. 11, 510–520. 10.1038/ncb0509-510 19404330

[B51] Mordue D. G. Desai N. Dustin M. Sibley L. D. (1999). Invasion by *Toxoplasma gondii* establishes a moving junction that selectively excludes host cell plasma membrane proteins on the basis of their membrane anchoring. J. Exp. Med. 190, 1783–1792. 10.1084/jem.190.12.1783 10601353 PMC2195726

[B52] Morisaki J. H. Heuser J. E. Sibley L. D. (1995). Invasion of *Toxoplasma gondii* occurs by active penetration of the host cell. J. Cell. Sci. 108(Pt 6), 2457–2464. 7673360 10.1242/jcs.108.6.2457

[B53] Ortega-Barria E. Boothroyd J. C. (1999). A Toxoplasma lectin like activity specific for sulfated polysaccharides is involved in host cell infection. J. Biol. Chem. 274, 1267–1276. 10.1074/jbc.274.3.1267 9880495

[B54] Podinovskaia M. Descoteaux A. (2015). *Leishmania* and the macrophage: a multifaceted interaction. Future Microbiol. 10, 111–129. 10.2217/fmb.14.103 25598341

[B55] Robertson M. J. Deane F. M. Stahlschmidt W. von Kleist L. Haucke V. Robinson P. J.. (2014). Synthesis of the Pitstop family of clathrin inhibitors. Nat. Protoc. 9, 1592–1606. 10.1038/nprot.2014.106 24922269

[B56] Rupper A. Lee K. Knecht D. Cardelli J. (2001). Sequential activities of phosphoinositide 3-kinase, PKB/Aakt, and Rab7 during macropinosome formation in *Dictyostelium*. Mol. Biol. Cell. 12, 2813–2824. 10.1091/mbc.12.9.2813 11553719 PMC59715

[B57] Sánchez E. Pérez-Núñez D. Revilla Y. (2017). Mechanisms of entry and endosomal pathway of african swine fever virus. Vaccines (Basel). 5:42. 10.3390/vaccines5040042 29117102 PMC5748609

[B58] Schnatwinkel C. Christoforidis S. Lindsay M. R. Uttenweiler-Joseph S. Wilm M. Parton R. G.. (2004). The rab 5 efector rabankirin-5 regulates anf coordinates different endocytic mechanisms. PLoS Biol. 2:E261. 10.1371/journal.pbio.0020261 15328530 PMC514490

[B59] Sibley L. D. (2011). Invasion and intracellular survival by protozoan parasites. Immunol. Rev. 240, 72–91. 10.1111/j.1600-065X.2010.00990.x 21349087 PMC3697736

[B60] Silva S. R. Meirelles S. S. De Souza W. (1982). Mechanism of entry of Toxoplasma gondii into vertebrate cells. J. Submicrosc. Cytol. 14, 471–482. 7175984

[B61] Sinai A. P. (2008). Biogenesis of and activities at the Toxoplasma gondii parasitophorous vacuole membrane. Subcell. Biochem. 47, 155–164. 10.1007/978-0-387-78267-6_12 18512349

[B62] Słońska A. Cymerys J. Bańbura M. W. (2016). Mechanisms of endocytosis utilized by viruses during infection. Postepy Hig. Med. Dosw. (Online). 70, 572–580. 10.5604/17322693.1203721 27333927

[B63] Tonkin M. L. Roques M. Lamarque M. H. Pugnière M. Douguet D. Crawford J.. (2011). Host cell invasion by apicomplexan parasites: insights from the co-structure of AMA1 with a RON2 peptide. Science 333, 463–467 10.1126/science.1204988 21778402

[B64] Vieira M. Dutra J. M. Carvalho T. M. Cunha-e-Silva N. L. Souto-Padrón T. Souza W. (2002). Cellular signaling during the macrophage invasion by *Trypanosoma cruzi*. Histochem. Cell. Biol. 118, 491–500. 10.1007/s00418-002-0477-0 12483314

[B65] Walker D. H. (2007). Rickettsiae and rickettsial infections: the current state of knowledge. Clin. Infect. Dis. 45(Suppl 1), S39–S44. 10.1086/518145 17582568

[B66] Walker D. M. Oghumu S. Gupta G. McGwire B. S. Drew M. E. Satoskar A. R. (2014). Mechanisms of cellular invasion by intracellular parasites. Cell. Mol. Life Sci. 71, 1245–1263. 10.1007/s00018-013-1491-1 24221133 PMC4107162

[B67] Wanderley J. L. Moreira M. E. Benjamin A. Bonomo A. C. Barcinski M. A. (2006). Mimicry of apoptotic cells by exposing phosphatidylserine participates in the establishment of amastigotes of *Leishmania* (L) *amazonensis* in mammalian hosts. J. Immunol. 176, 1834–1839. 10.4049/jimmunol.176.3.1834 16424214

[B68] Watarai M. Derre I. Kirby J. Growney J. D. Dietrich W. F. Isberg R. R. (2001). *Legionella pneumophila* is internalized by a macropinocytotic uptake pathway controlled by the Dot/Icm system and the mouse Lgn1 locus. J. Exp. Med. 194, 1081–1096. 10.1084/jem.194.8.1081 11602638 PMC2193510

[B69] Zhou W. Quan J. H. Lee Y. H. Shin D. W. Cha G. H. (2013). Toxoplasma gondii proliferation require down-regulation of host Nox4 expression via activation of PI3 Kinase/Akt signaling pathway. PLoS One. 8:e66306. 10.1371/journal.pone.0066306 23824914 PMC3688893

[B70] Zhu W. Li J. Pappoe F. Shen J. Yu L. (2019). Strategies developed by *Toxoplasma gondii* to survive in the host. Front. Microbiol. 10:899. 10.3389/fmicb.2019.00899 31080445 PMC6497798

